# One-pot biosynthesis of *7β*-hydroxyandrost-4-ene-3,17-dione from phytosterols by cofactor regeneration system in engineered *mycolicibacterium neoaurum*

**DOI:** 10.1186/s12934-022-01786-5

**Published:** 2022-04-09

**Authors:** Yun-Qiu Zhao, Yong-Jun Liu, Wei-Ting Ji, Kun Liu, Bei Gao, Xin-Yi Tao, Ming Zhao, Feng-Qing Wang, Dong-Zhi Wei

**Affiliations:** State Key Laboratory of Bioreactor Engineering, Newworld Institute of Biotechnology, East China University of Science and Technology, Shanghai, 200237 China

**Keywords:** *7β*-hydroxyandrost-4-ene-3,17-dione, Phytosterols, mP450-BM3, Nicotinamide adenine dinucleotide phosphate regeneration, *Mycolicibacterium*

## Abstract

**Background:**

*7β*-hydroxylated steroids (7β-OHSt) possess significant activities in anti-inflammatory and neuroprotection, and some of them have been widely used in clinics. However, the production of 7β-OHSt is still a challenge due to the lack of cheap *7β*-hydroxy precursor and the difficulty in regio- and stereo-selectively hydroxylation at the inert C7 site of steroids in industry. The conversion of phytosterols by *Mycolicibacterium* species to the commercial precursor, androst-4-ene-3,17-dione (AD), is one of the basic ways to produce different steroids. This study presents a way to produce a basic *7β*-hydroxy precursor, *7β*-hydroxyandrost-4-ene-3,17-dione (7β-OH-AD) in *Mycolicibacterium*, for 7β-OHSt synthesis.

**Results:**

A mutant of P450-BM3, mP450-BM3, was mutated and engineered into an AD producing strain for the efficient production of 7β-OH-AD. The enzyme activity of mP450-BM3 was then increased by 1.38 times through protein engineering and the yield of 7β-OH-AD was increased from 34.24 mg L^− 1^ to 66.25 mg L^− 1^. To further enhance the performance of 7β-OH-AD producing strain, the regeneration of nicotinamide adenine dinucleotide phosphate (NADPH) for the activity of mP450-BM3-0 was optimized by introducing an NAD kinase (NADK) and a glucose-6-phosphate dehydrogenase (G6PDH). Finally, the engineered strain could produce 164.52 mg L^− 1^ 7β-OH-AD in the cofactor recycling and regeneration system.

**Conclusions:**

This was the first report on the one-pot biosynthesis of 7β-OH-AD from the conversion of cheap phytosterols by an engineered microorganism, and the yield was significantly increased through the mutation of mP450-BM3 combined with overexpression of NADK and G6PDH. The present strategy may be developed as a basic industrial pathway for the commercial production of high value products from cheap raw materials.

**Supplementary Information:**

The online version contains supplementary material available at 10.1186/s12934-022-01786-5.

## Background

 As a class of drugs with diverse therapeutic activities on reproductive health, endocrine dyscrasia, inflammation, and so on, steroids are in great demand in the global market [1, 2]. Among them, some *7β*-hydroxylated steroids (7β-OHSt) and corresponding derivatives can be used to treat inflammation, cholestasis, and chronic neuronal damage [3–5]. However, the production of steroidal C7β alcohols is still a challenge due to the lack of cheap *7β*-hydroxy precursor. For most of the common steroidal molecules, C7 is usually not an active site and thus the hydroxylation at C7β in a regio- and stereo-selective way is not easily accessible in the industry [6, 7]. Therefore, it is necessary to develop a feasible way to produce commodifiable and universal 7β-hydroxylated synthons for the synthesis of 7β-OHSt.

The *7β*-hydroxylated derivative of androst-4-ene-3,17-dione (AD) has been recognized as a favorable synthon for the synthesis of 7β-OHSt, such as ursodeoxycholic acid (UDCA) [8, 9]. However, the selective *7β*-hydroxylation of AD by chemical reaction is not easy and seldom industrially applied due to the low regio- and stereo-selectivity [10, 11]. For the hydroxylation of steroids, biotransformation has been widely accepted as an available way due to the comparatively higher stereo-selectivity [12, 13], and some microbial hydroxylation methods, such as *11α*- and *11β*-hydorxylation, have already been industrially applied for decades [14, 15]. Up to now, some filamentous fungi that can hydroxylate steroids at C7β have been screened to allow the production of 7β-OHSt by microbial transformations [16–18]. However, these strains have not been applied in the industry because of the insufficient regio- and stereo-selectivities and low conversion rates. Moreover, due to the difficulty in genetic modification of these uncommon fungi, it is still a challenge to engineer these strains for the efficient production of 7β-OHSt.

P450-BM3 is a well-known hydroxylase of fatty acids from *Bacillus megaterium* and one of the most active cytochrome P450 monooxygenases (CYPs) due to its self-sufficient character in electron transfer [19, 20]. Therefore, it is a promising biocatalyst for steroid hydroxylation in protein engineering and some mutants of P450-BM3 have been generated to introduce hydroxyl group selectively at different sites of the A or D ring of steroids [21, 22]. Recently, a mutant of P450-BM3 (mP450-BM3) with a remarkable *7β*-hydroxylation activity on AD has been reported and brings out a new chance to develop engineered microorganisms to produce *7β*-hydroxylated synthon 7β-OH-AD for the synthesis of 7β-OHSt [23].

Nowadays, plentiful cheap phytosterols (PS), extracted from vegetable oil deodorizer distillate and wood pulp tall oil, have been used as the main raw materials for production of most of synthetic steroids in the industry via the microbial conversion of PS to some key synthons, especially AD [24, 25]. Although a cascade process can be used to produce *7β*-hydroxylated AD (7β-OH-AD) from PS by the conversion in engineered *Mycolicibacterium* sp. and the biocatalytic reaction mediated by mP450-BM3, a simple one-pot biotransformation system for the production of 7β-OH-AD from PS can be achieved by engineering mP450-BM3 into *Mycolicibacterium* sp. P450-BM3 is an NADPH-dependent CYP, and the availability of NADPH determines its catalytic efficiency. Thus, the supply of NADPH is often a key limiting factor of the activity of P450-BM3 [26, 27]. During the conversion process of PS to AD, NADH instead of NADPH is generated as the main cofactor [28]. Zhao et al. revealed that the lack of NADPH was a key rate-limiting factor of the activity of the heterologous NADPH-dependent enzyme expressed in *Mycolicibacterium neoaurum* (*M. neoaurum*) for the conversion of AD to 5α-AD, and that enhancing the NADPH/NADP^+^ ratio can efficiently increase the yield of 5α-AD by 28% [29]. Therefore, to achieve an optimal activity of mP450-BM3 in *Mycolicibacterium* sp., an adequate supply of NADPH needs to be guaranteed.

**Fig. 1:**
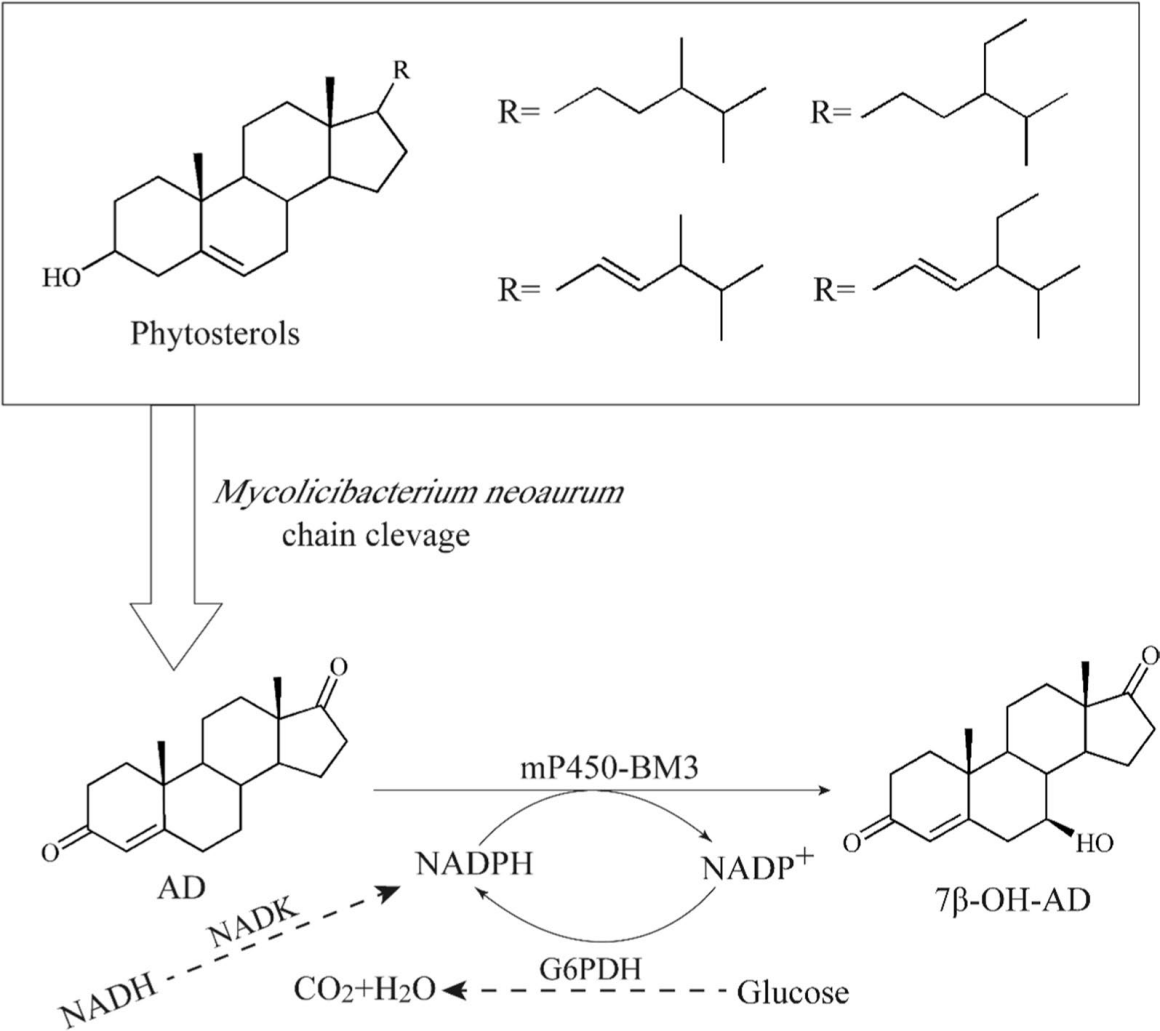
The production of 7β-OH-AD by recombinant *Mycolicibacterium neoaurum* strains with the NADPH regeneration system

In this study, to develop a cheap way to produce a universal synthon for 7β-OHSt production, a 7β-OH-AD producing strain was constructed by modifying the metabolic pathway of PS and introducing mP450-BM3 into the AD producing strain. In view of the insufficient activity of mP450-BM3 caused by the lack of NADPH, a combined strategy to enhance the amount of NADPH and the ratio of NADPH/NADP^+^ was performed. Firstly, the superfluous NADH generated during the conversion of PS to AD was transformed into NADPH by augmenting the expression of NAD kinase. Next, the NADPH-regenerating glucose-6-phosphate dehydrogenase (G6PDH) was over-expressed in *M. neoaurum* to cycle the regeneration of NADP^+^ to NADPH. Finally, a one-pot biocatalytic process of PS to 7β-OH-AD was successfully achieved (Fig. 1). To our knowledge, this was the first report to directly synthesize 7β-OH-AD from the commonly used raw material PS with a one-pot biocatalytic route. The study provides a promising way to produce 7β-OHSt via the universal synthon 7β-OH-AD in the pharmaceutical industry.

## Results

### Construction of the one-pot conversion pathway from PS to 7β-OH-AD in *M. neoaurum*

The conversion of PS to AD by *Mycolicibacteria* is a basic way to supply the universal synthon for production of diverse steroids by further structure modification. To simplify the conventional way to produce 7β-OH-AD, here, an integrated microbial cell factory was developed to directly convert PS to 7β-OH-AD in one-pot. Therefore, M3 derived from *M. neoaurum* ATCC 25,795 by deleting all the characterized 3-ketosteroid-Δ^1^-dehydrogenase (*kstD1*, *kstD2*, and *kstD3*) and the 3-ketosteroid-*9α*-hydroxylase isoenzymes (*kshA1* and *kshA2*), which can effectively transform PS to AD, was selected as a chassis to construct a 7β-OH-AD producing strain [30]. According to the reported mutant of P450-BM3 with *7β*-hydroxylation activity to AD (mP450-BM3), the codon-optimized genes of P450-BM3 and mP450-BM3 were firstly introduced into strain M3 by an episomal plasmid pMV261 and verified by colony PCR and enzyme digestion techniques (Additional file 1: Fig. S1), thus resulting in the recombinant strains M3-*BM3* and M3-*mBM3* (Table 1). Compared to M3-261 and M3-*BM3*, the strain M3-*mBM3* showed a firm activity in the conversion of PS and AD and generated an extra product, which appeared in the position of standard 7β-OH-AD on the thin layer chromatography (TLC) plate (Additional file 1: Fig. S2). High resolution mass spectrometry (HRMS) analysis showed that the molecular weight of the purified product was 302.1954 m/z (Additional file 1: Fig. S3A), which was the same as that of standard 7β-OH-AD [25, 26]. High performance liquid chromatography (HPLC) showed that the extra product was consistent with the standard 7β-OH-AD in the peak time (Additional file 1: Fig. S3B). These results indicated that a 7β-OH-AD producing strain was successfully constructed. The *7β*-hydroxylase activity in the M3-*mBM3* was determined to be 1.42 ± 0.13 U g^− 1^, whereas no *7β*-hydroxylase activity was observed in M3-261 and M3-*BM3* (Table 2).

**Table 1:** Strains and plasmids used in this study

Name	Description	Sources
Strains		
* Escherichia coli* DH 5α	*E. coli* cloning host	Transgen Biotech
* Mycolicibacterium neoaurum*	Source of G6PDH and NADK genes	This lab
M3	*Ksdd* and *KshA* deletion mutant of *MNR* ATCC 25,795	This lab
M3-261	M3 containing pMV261 as control	This study
M3-*BM3*	M3 expressing original P450-BM3 gene	This study
M3*-mBM3*	M3 expressing mP450-BM3 gene (F87A/T260G)	This study
M3*-mBM3-0*	M3 expressing mP450-BM3-0 gene (S72W/V78L/A82L	This study
	/T88S/A328G/A330W)	
M3*-mBM3-0-NADK2*	M3 expressing mP450-BM3-0 and *NADK2* genes	This study
M3*-mBM3-0-G6PDH2*	M3 expressing mP450-BM3-0 and *G6PDH2* genes	This study
M3*-mBM3-0*-*NADK2-G6PDH2*	M3 expressing mP450-BM3-0, *NADK2* and *G6PDH2* genes	This study
Plasmids		
pUC57-*BM3*	The codon-optimized original P450-BM3 gene delivered	Shanghai Generay
	by pUC57, Amp^R^	Biotech Co. Ltd
pUC57-*mBM3*	The codon-optimized mP450-BM3 gene delivered by	Shanghai Generay
	pUC57, Amp^R^	Biotech Co. Ltd
pMV261	Shuttle vector of *Mycobacterium* and *E. coli*, P_*hsp60*_, Kan^R^	Dr. W. R. Jacobs Jr. for
		providing pMV261
pMV261*-BM3*	pMV261 containing original P450-BM3 gene, Kan^R^	This study
pMV261*-mBM3*	pMV261 containing mP450-BM3 gene, Kan^R^	This study
pMV261*-mBM3*-*0*	pMV261 containing mP450-BM3-0 gene, Kan^R^	This study
pMV261*-NADK*	pMV261 containing NADK gene from *M. neoaurum*, Kan^R^	This study
pMV261*-G6PDH*	pMV261 containing G6PDH gene from *M. neoaurum*, Kan^R^	This study
pMV261*-mBM3*-*0*-*NADK2*	pMV261 containing mP450-BM3-0 gene and NADK2	This study
	Gene from *M. neoaurum*, Kan^R^	
pMV261*-mBM3*-*0*-*G6PDH2*	pMV261 containing mP450-BM3-0 gene and G6PDH2	This study
	Gene from *M. neoaurum*, Kan^R^	
pMV261*-mBM3*-*0*-*NADK2-*	pMV261 containing mP450-BM3-0 gene, NADK2 and	This study
* G6PDH2*	G6PDH2 genes from *M. neoaurum*, Kan^R^	

Amp^R^: ampicillin-resistant, Kan^R^: kanamycin-resistant

**Table 2:** The specific activities of 7β-hydroxylase, NADK and G6PDH in recombinant *Mycolicibacterium neoaurum* strains

Strains	Enzyme activity		
	7β-hydroxylase	NADK	G6PDH
	(U g^− 1^)	(U g^− 1^)	(U g^− 1^)
M3-261	0	1.24 ± 0.06	1.37 ± 0.07
M3-*BM3*	0	1.17 ± 0.04	1.31 ± 0.12
M3-*mBM3*	1.42 ± 0.13	1.12 ± 0.07	1.25 ± 0.04
M3-*mBM3*-*0*	2.34 ± 0.11	1.08 ± 0.09	1.18 ± 0.05
M3-*mBM3*-*0-NADK2*	2.57 ± 0.08	2.56 ± 0.12	1.21 ± 0.15
M3-*mBM3*-*0-G6PDH2*	2.72 ± 0.12	1.24 ± 0.05	3.42 ± 0.13
M3-*mBM3*-*0-NADK2-G6PDH2*	3.03 ± 0.06	2.35 ± 0.15	3.34 ± 0.09

With PS and AD as substrates, M3-*mBM3* generated 34.24 and 47.39 mg L^− 1^ 7β-OH-AD, respectively. The conversion capacity of PS to AD was higher, but a large quantity of AD was not converted into 7β-OH-AD (Additional file 1: Fig. S4). The results indicated that the enzymatic activity of mP450-BM3 in M3-*mBM3* was much lower than that in the *Escherichia coli* [23]. Given that it was expressed by a high-copy plasmid pMV261 with a powerful promoter P_*hsp60*_ in M3-*mBM3*, the expression level of mP450-BM3 might be a key factor limiting its activity. Therefore, two factors closely related to the low expression level of mP450-BM3 in M3-mBM3 were further studied. Firstly, the specific activity of mP450-BM3 in M3-*mBM3* was not high enough and further evolution might boost its activity. Secondly, NADPH in M3-*mBM3* was not enough for mP450-BM3 because the catabolic process of PS to AD generated NADH other than NADPH [28, 31].

### Evolving the activity of *7β*-hydroxylase by site-specific mutagenesis

**Fig. 2:**
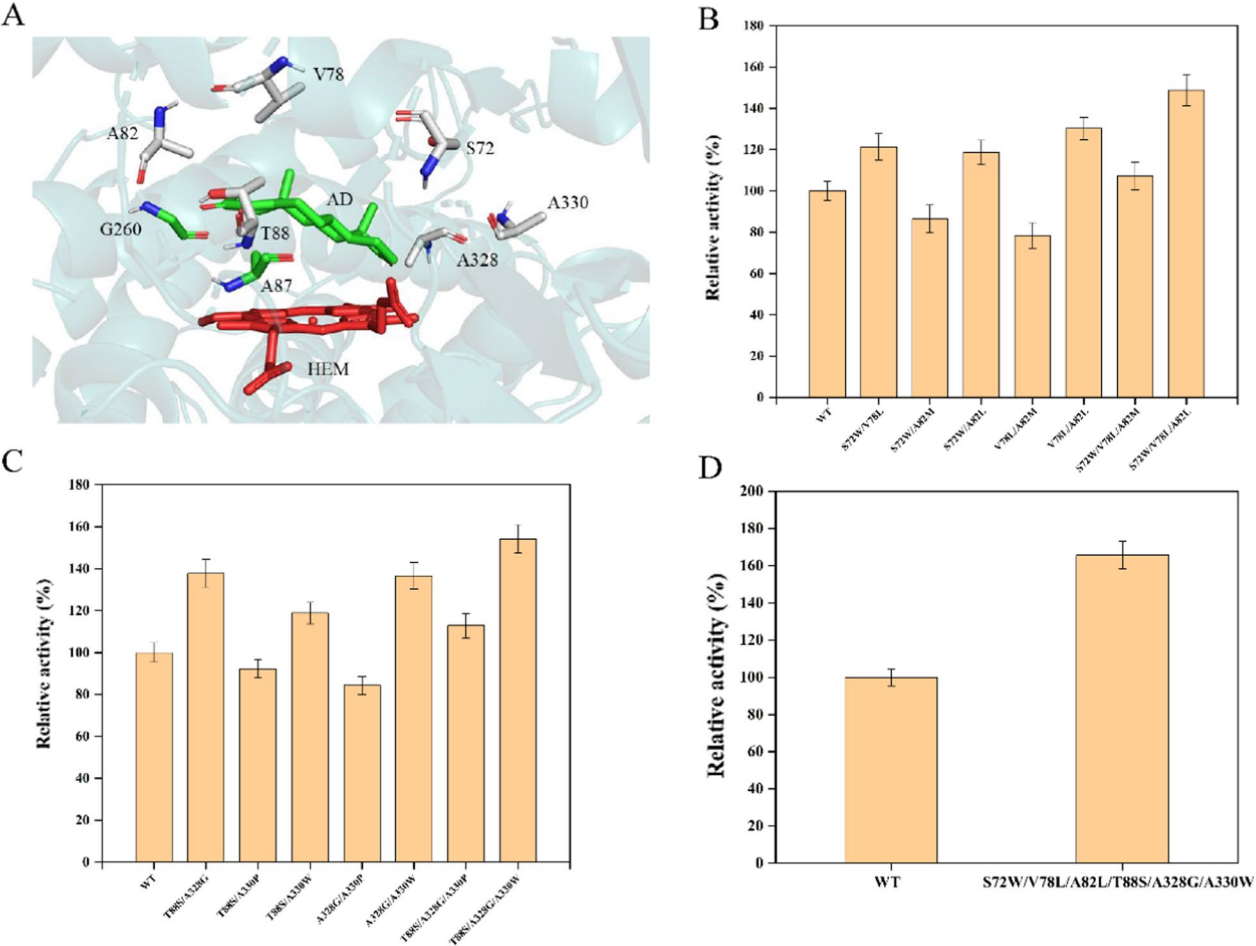
Rational design of the mP450-BM3 to improve its activity to AD. **A** Docking of the ligand AD into the mP450-BM3 crystal structure. AD is shown in green, heme is shown in red. **B** Relative activity of mP450-BM3 (WT) and mutants (away from the heme) to AD. **C** Relative activity of mP450-BM3 (WT) and mutants (close to the heme) to AD. **D** Relative activity of mP450-BM3 (WT) and the mutant (S72W/V78L/A82L/T88S/A328G/A330W) to AD. All assays were performed in triplicate with three independent measurements. Standard deviations of the biological replicates are represented by error bars

It was considered a feasible method for promoting protein catalytic performance by semi-rational design and molecular remodeling. In order to elucidate the interaction of mP450-BM3 with substrate AD, a homology model was constructed by using NPG-P450-BM3 (PDB ID:4kpa) as the initial search model. The combined mutation of F87A and T260G in P450-BM3 (mP450-BM3) generated a new product, 7β-OH-AD [22]. Therefore, this mutant was used as the starting template for further mutagenesis (Fig. 2A).

The following active sites residues (S72, V78, A82, T88, A328, A330) were subjected to saturation mutagenesis, and the results showed that S72W, V78L, A82M, A82L, T88S, A328G, A330W and A330P mutants resulted in higher activities than the wild type (mP450-BM3) (Fig. S5). Afterward, the variants were divided into two groups based on the distance from the heme. The amino acid residues distant from the heme (Ser72, Val78 and Ala82) were mutated in combination to construct double and triple mutants. Among these mutants, the resulting S72W/V78L/A82L achieved the highest reactivity (Fig. 2B). Similarly, the other double and triple mutants were constructed based on the mutants (T88S, A328G, A330W and A330P) that have mutation in the residues close to the heme, and among which the best *7β*-hydroxylation activity to substrate AD was observed in the mutant T88S/A328G/A330W (Fig. 2C). Mutation of these residues altered the size and shape of the substrate binding pocket. For example, polar residue Thr88 located in the substrate-binding pocket was mutated to serine, which removed the steric hindrance of the methyl groups between Ala87 and Thr88, ensuring that AD could be oriented along the B’C loop. The mutation of A82L increased the space of active site and made a conformational change in the mP450-BM3, thus enabling the oxidation of omeprazole at the 7-methyl position. Finally, a combinatorial mutation between S72W/V78L/A82L and T88S/A328G/A330W was carried out. Fortunately, but not surprisingly, the highest activity was achieved in the mutant S72W/V78L/A82L/T88S/A328G/A330W (mP450-BM3-0, Fig. 2D). The 7β-hydroxylase activity in the mutant (mP450-BM3-0) reached to 2.34 ± 0.11 U g^− 1^ (Table 2), and the yield of 7β-OH-AD increased to 66.25 ± 2.42 mg L^− 1^ (Table 3). In addition, the stereoselectivity was also improved to a certain extent (Additional file 1: Fig. S3B).

**Table 3:** The durations and titer of PS conversion by different recombinant *Mycolicibacterium neoaurum* strains

Strains	Durations (d)	Titer (mg L^− 1^)
M3-261	–	0
M3-*BM3*	–	0
M3-*mBM3*	7	34.24 ± 1.34
M3-*mBM3*-*0*	7	66.25 ± 2.42
M3-*mBM3*-*0*-*NADK2*	6	94.63 ± 2.27
M3-*mBM3*-*0-G6PDH2*	6	117.46 ± 1.49
M3-*mBM3*-*0-NADK2-G6PDH2*	6	139.87 ± 3.73

### Regeneration and balance of cofactors in the conversion of PS to 7β-OH-AD

**Fig. 3:**
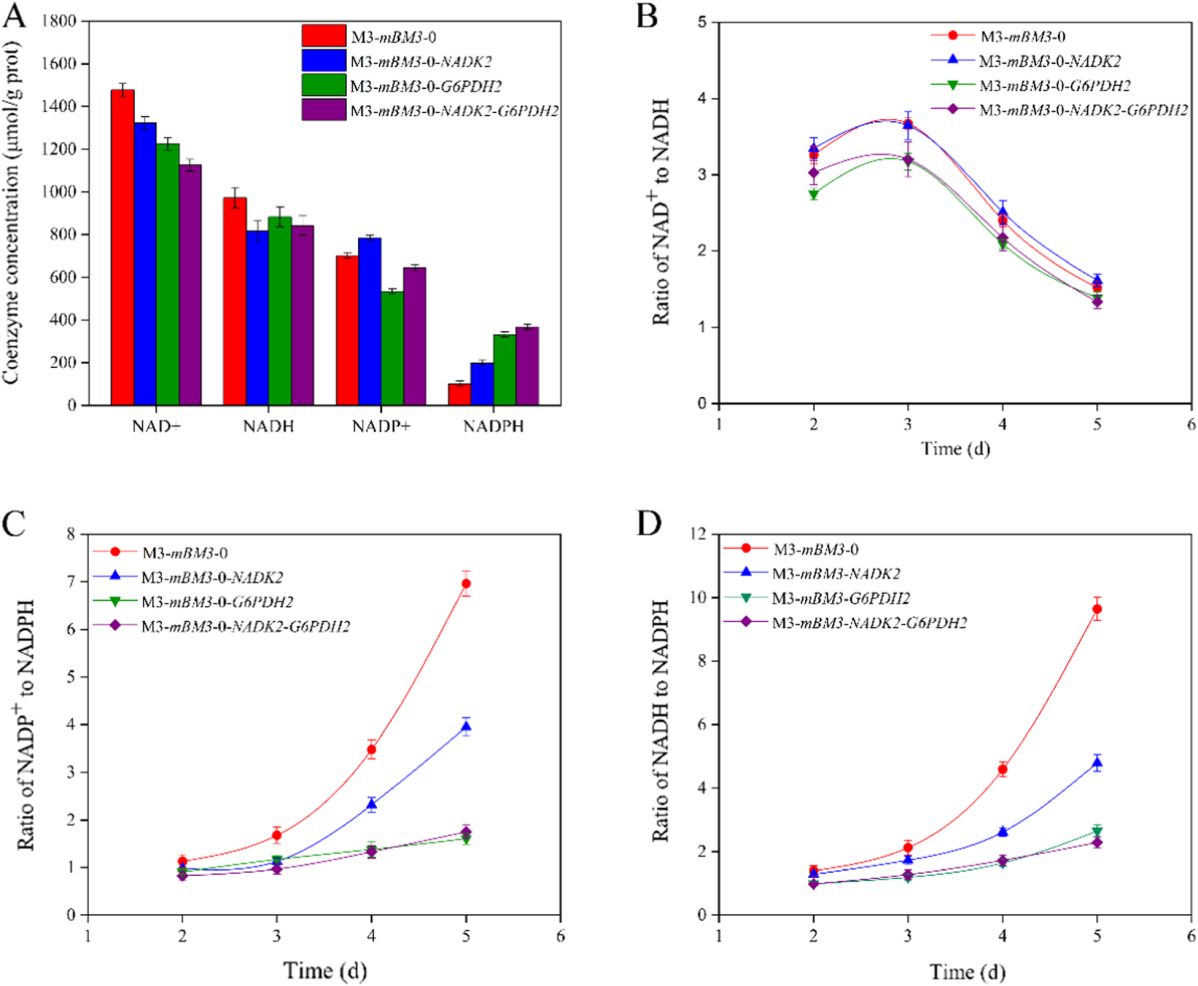
The concentration and ratio of coenzymes in different recombinant *Mycolicibacterium neoaurum* strains. **A** The different coenzyme concentration on the 5th day in recombinant *M. neoaurum* strains. **B** The ratio of NAD^+^ to NADH in recombinant *M. neoaurum* strains. **C** The ratio of NADP^+^ to NADPH in recombinant *M. neoaurum* strains. **D** The ratio of NADH to NADPH in recombinant *M. neoaurum* strains. All assays were performed in triplicate with three independent measurements. Standard deviations of the biological replicates are represented by error bars

As an NADPH-dependent enzyme, mP450-BM3-0 requires the participation of cofactor NADPH to convert AD into 7β-OH-AD. It was found that the content of NADPH in M3-*mBM3*-0 was much less than that of NADH on the fifth day (Fig. 3A), the ratio of NADH/NADPH and NADP^+^/NADPH sharply increased along with the conversion of PS to 7β-OH-AD (Fig. 3B, C). These data demonstrated that the lack of NADPH supply caused by the low content of NADPH and the frustrated regeneration of NADPH was responsible for the low activity of the mutants of P450-BM3 in the engineered 7β-OH-AD strains. To enhance the supply of NADPH, therefore, a combined strategy was adopted to increase the content of NADPH.

**Fig. 4:**
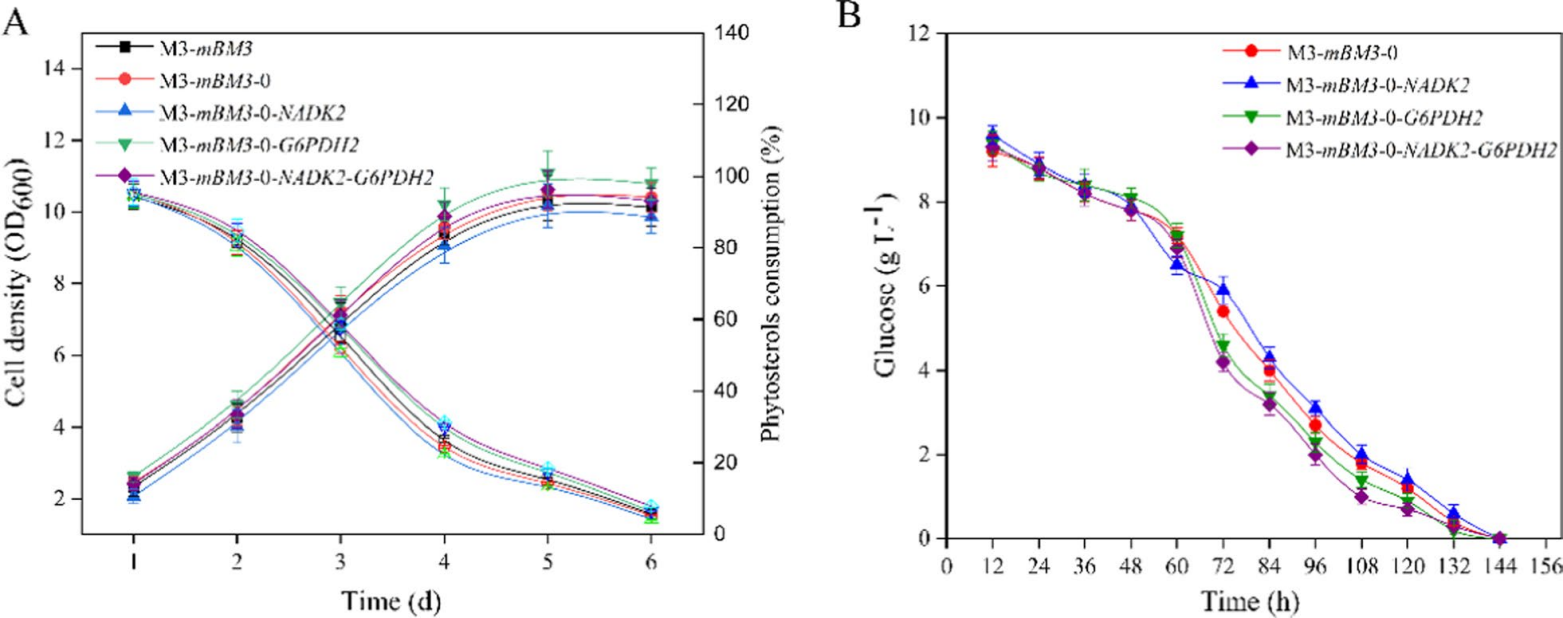
Growth and metabolism statues of different recombinant strains. **A** The growth (closed square) and phytosterols consumption (open square) of recombinant strains. **B** The glucose consumption of recombinant strains. All assays were performed in triplicate with three independent measurements. Standard deviations of the biological replicates are represented by error bars

Twenty-one mole NADH would be generated during the conversion of 1 mol PS into AD, and reducing the ratio NADH/NAD^+^ has been demonstrated to boost the conversion of PS to AD [28]. Therefore, the transformation of NADH to NADPH may promote the conversion of PS to 7β-OH-AD via AD. The strain M3-*mBM3*-*0*-*NADK2* was obtained by augmenting the gene NADK in strain M3-*mBM3*-*0*, which could convert partial NAD(H) into NADP(H). The NADK enzyme activity in M3-*mBM3-0*-*NADK2* was more than 2 times that in M3-*mBM3-0* (Table 2). The content of NADPH increased from 100.79 µM to 298.53 µM on the 5th day, whereas the content of NADH was significantly reduced in the conversion process of PS (Fig. 3A). In addition, the augmentation of NADK in M3-*mBM3*-*0*-*NADK2* showed no effects on the ratio of NAD^+^/NADH in the conversion process of PS to 7β-OH-AD (Fig. 3C), but it profoundly increased the ratio of NADPH/NADP^+^ in the conversion process of PS, especially in the later stages (Fig. 3D). These data fully demonstrated that converting a small part of NAD(H) to NADP(H) was an effective approach to enhance the supply of NADPH in the conversion process of PS to 7β-OH-AD. The approach could increase both the content of NADP(H) and the ratio of NADPH/NADP^+^ without a negative effect on cell growth and the conversion of PS (Fig. 4A). As expected, therefore, the titer of 7β-OH-AD was significantly enhanced by 30.01% (Additional file 1: Fig. S4B).

It was found that abundant NADP^+^ was generated during the conversion from PS to 7β-OH-AD, accompanied by rapid depletion of NADPH (Fig. S6), resulting in the ratio of NADP^+^/NADPH increased continuously, which indicated that regeneration rate of NADPH from NADP^+^ was slow in M3-*mBM3-0* (Fig. 3D). Lee et al. had enhanced the production of *ɛ*-caprolactone by overexpressing an NADPH-regenerating glucose-6-phosphate dehydrogenase in recombinant *E. coli* [32]. In order to promote the cyclic regeneration of NADPH from NADP^+^, a native NADP^+^ dependent G6PDH from *M. neoaurum* was expressed in strain M3-*mBM3*-*0* to generate strain M3-*mBM3*-*0*-*G6PDH2*, which could increase G6PDH activity by 2.9-folds (Table 2). As expected, the ratio of NADP^+^/NADPH was significantly reduced in the conversion process of PS, displaying the augmentation effect on NADK (Fig. 3D), though the ratio of NAD^+^/NADH was unexpectedly significantly reduced due to the side effect of G6PDH on the conversion of NAD^+^ to NADH (Fig. 3C). In contrast to that of strain M3-*mBM3*-*0*, the 7β-OH-AD production titer of M3-*mBM3*-*0*-*G6PDH2* was enhanced by 43.68% and only slight negative effects on cell growth and PS conversion were observed (Fig. 4A). Therefore, increasing the cyclic regeneration of NADPH by the expression of G6PDH could also boost the activity of *7β*-hydroxylase for the conversion of AD to 7β-OH-AD.

The co-expression of NADK and G6PDH was subsequently preformed in strain M3-*mBM3*-*0* to generate strain M3-*mBM3*-*0*-*NADK2*-*G6PDH2*, which showed a similar growth phenotype to strain M3-*mBM3*-*0* (Fig. 4A). The combined expression of both NADK2 and G6PDH2 resulted in an increased content of NADPH (Fig. 3A), a reduced ratio of NAD^+^/NADH (Fig. 3C), and an enhanced ratio of NADPH/NADP^+^ (Fig. 3D).

Finally, due to the significant increase in NADPH supply, the strain accordingly achieved a further increase in the production of 7β-OH-AD, 139.87 mg L^− 1^, which was 52.7% higher than that in M3-*mBM3*-*0*.The above results confirmed that the lack of NADPH supply was a key limiting factor in the conversion of PS to 7β-OH-AD. Transforming part of abundant NADH generated in the PS conversion process to NADPH and promoting the cyclic regeneration of NADPH could boost the production of 7β-OH-AD without affecting the growth of engineered strains and the conversion performance of PS.

### Optimization of conversion conditions of PS to 7β-OH-AD

The biotransformation of PS to steroidal synthons by *Mycolicibacterium* sp. is a co-metabolism process with glucose and PS as well as its main metabolites are hydrophobic. The uncommon traits make the conversion of PS to 7β-OH-AD be more complex in fermentation conditions. Therefore, some key factors affecting the conversion of PS to 7β-OH-AD were further investigated in order to enhance its application potential.

**Fig. 5:**
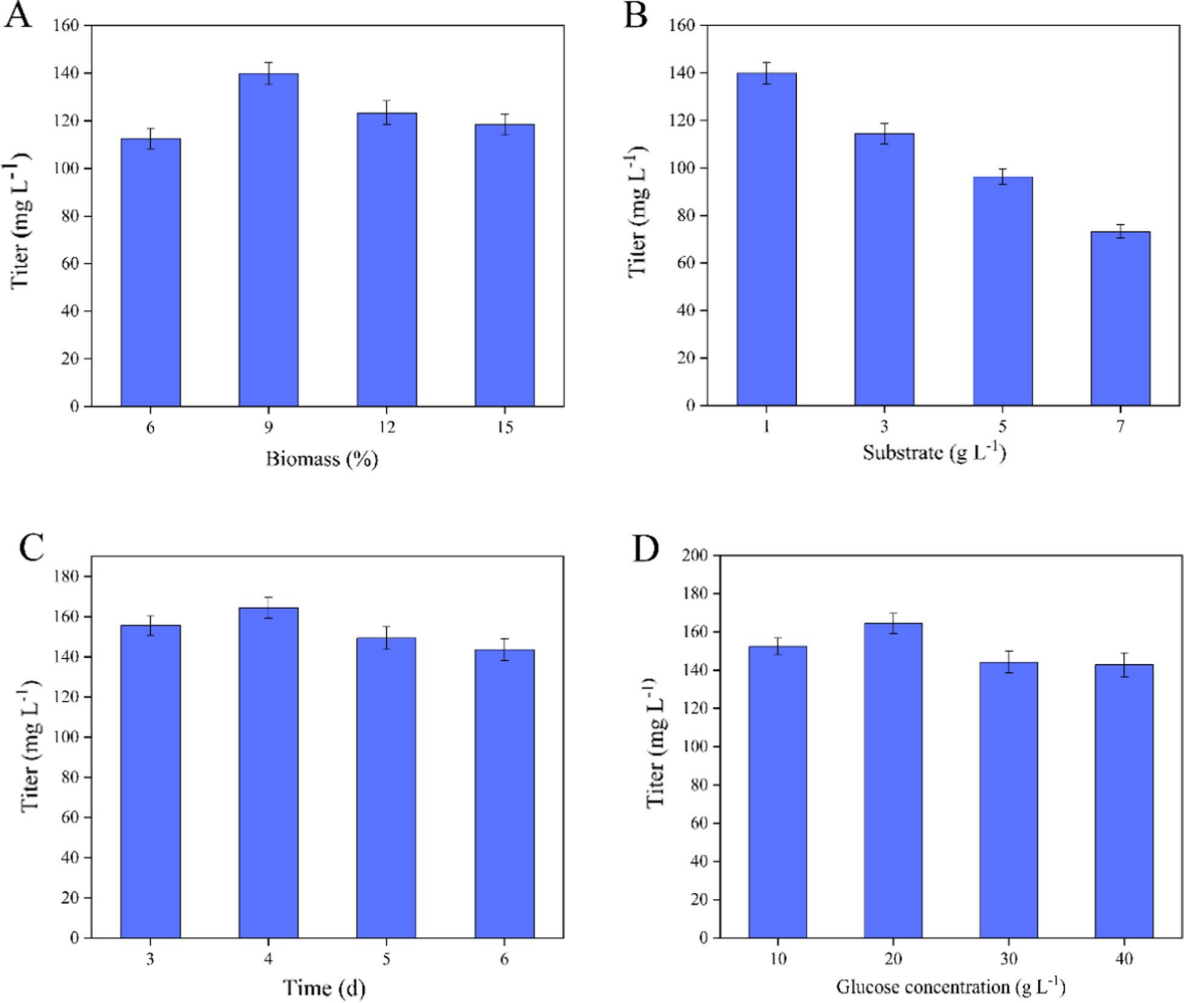
The effect of different fermentation conditions on production of 7β-OH-AD. **A** Effects of different biomass of M3-*mBM3*-*0*-*NADK2-G6PDH2* (6%, 9%, 12%, 15%) on 7β-OH-AD production. **B** Effects of different substrate concentrations (1 g L^− 1^, 3 g L^− 1^, 5 g L^− 1^, 7 g L^− 1^) on the conversion rate. **C** Effects of adding time of glucose (3 d, 4 d, 5 d, 6 d) on conversion rate. **D** Effects of concentrations of glucose supplement (10 g L^− 1^, 20 g L^− 1^, 30 g L^− 1^, 40 g L^− 1^) on conversion rate. All assays were performed in triplicate with three independent measurements. Standard deviations of the biological replicates are represented by error bars

*Mycolicibacterium* species experience complex physiological and morphologic changes along with the consumption of nutrition ingredients and their generation period is generally longer than that of common microorganisms [33]. Thus, the growth status was closely related to the conversion capacity of PS. Nevertheless, it was difficult to precisely determine the growth curve of *Mycolicibacteria* as PS and its metabolites were dissolved in the fermentation process. Therefore, the initial inoculation dosage of the fermentation process was investigated. The result indicated that 9% inoculation dosage with pre-cultured cells (OD_600_ value of 3.0) in the logarithmic phase as seeds was the optimal dosage for production of 7β-OH-AD and the further increase in the inoculation dosage was no more conducive to the production of 7β-OH-AD (Fig. 5A).

The conversion process of steroids as well as PS in an aqueous fermentation system is usually recognized as a pseudo-crystalline fermentation process and the solubility or dispersion of PS is the key factor limiting its conversion efficiency. PS can be partially dissolved in some co-solvents, such as tween, ethanol, and cyclodextrin. However, excessive substrates are difficult to be dissolved and dispersed and have an inhibitory effect on strains, thus resulting in low cell viability and conversion. Therefore, it is crucial to select a suitable substrate concentration for the conversion of PS into 7β-OH-AD. The conversion efficiency decreased gradually with the increase of substrate concentration (Fig. 5B). The titer of 7β-OH-AD was only 73.28 mg L^− 1^ when the substrate concentration reached 7 g L^− 1^. The low substrate concentration may be due to the restriction of activity of mP450-BM3, which limited its use of the substrates PS. The viscosity of the solution increased with the increase of substrate concentration, thus affecting the transfer rate of oxygen [34]. Thus, some efforts such as DNA shuffling and oxygen improvement should be made to increase the activity of mP450-BM3 for further improving conversion efficiency.

Glucose can not only be metabolized with sterols to support the growth of strains, but also can used as a co-substrate for the regeneration of NADPH. Therefore, it is necessary to explore the concentration and feeding time of glucose. The consumption rate of glucose increased significantly on the third day, whereas the recombinant strains grew rapidly (Fig. 4). Therefore, it was proper to add glucose after the third day to ensure the optimal conditions for the growth and metabolism of recombinant strains. The highest conversion efficiency was reached when glucose was added on the fourth day (Fig. 5C). Most of glucose was consumed after the fourth day, thus leading to the declined efficiency in the later fermentation stage.

A suitable concentration of glucose is also a crucial factor for the conversion. The conversion was improved by the increased glucose concentration, and the highest conversion efficiency was reached when the supplement concentration of glucose was 20 g L^− 1^ (Fig. 5D). When the concentration of glucose was further increased, the conversion efficiency was no longer increased. The state of fermentation broth would be changed by excessive glucose, thus adversely affecting the accumulation of fermentation products.

## Discussion

Steroids at *7β*-hydroxylation and their derivatives have good neuroprotective and anti-inflammatory activities, which have been widely used in the treatment of chronic neuronal damage [3, 4]. However, it is difficult to develop a method for *7β*-hydroxylation of steroids by microorganisms due to its low stereoselectivity [6, 10]. Li and Bornscheuer et al. have obtained the mutants capable of highly regio- and stereoselective *7β*-hydroxylation of steroids by semi-rational design of cytochrome P450 [23, 35]. However, it was not practical to apply the above catalytic reaction to industrial production due to the expensive substrates. *Mycolicibacterium* can produce steroid precurosrs via the nucleus oxidation and the side chain degradation of PS, a cheap and available industrial waste [36, 37]. The biotransformation from PS to AD was carried out by multi-catabolic enzymes, and the accumulation of AD in *Mycolicibacterium* can be realized by the method of metabolic engineering [30, 38]. In this study, the mP450-BM3 capable of *7β*-hydroxylation to steroids was selected and expressed in M3, an AD-producing strain, to realize the one-pot biosynthesis of 7β-OH-AD from biology waste PS (Fig. 1).

Analysis of fermentation products showed that amount of intermediate AD was present, while the titer of product 7β-OH-AD was not high, ruling out the possibility that the low yield of product was caused by the insufficient intermediate (Additional file 1: Fig. S4A). The low expression level of P450 in microorganisms limited its application in biosynthesis of many natural products [39, 40]. It was an effective approach to improve the biotransformation efficiency of AD to 7β-OH-AD by optimizing the expression of mP450-BM3. Semi-rational design and molecular remodeling are feasible methods for promoting the catalytic performance of P450 [41, 42]. The optimization by saturation mutagenesis and combined mutation have successfully improved the *7β*-hydroxylation activity of mP450-BM3 towards AD, and the yield of the 7β-OH-AD was increased as well (Table 2 and Additional file 1: Fig. S4B).

Cofactor engineering has been widely used to promote production of important primary and secondary metabolites [43–45]. Given the multi-catabolic enzymes biotransformation of phytosterols into AD, cofactors are closely related to the PS side-chain degradation [46]. 21 mol NADH would be generated during the conversion of one mole PS into AD [47]. NADPH was consumed continuously during the process of AD to 7β-OH-AD catalyzed by mP450-BM3-0, accompanied by massive generation of NADP^+^ (Fig. 3). Therefore, the balance between intracellular coenzyme I and II, or the oxidized and reduced coenzyme has been a key factor in regulating the catalytic process of PS to 7β-OH-AD. The overexpression of NADK and G6PDH in *Mycolicibacterium* increased the content of NADPH and regulated the balance of intracellular coenzyme successfully. Furthermore, mP450-BM3-0 can fully perform its function in the NADPH regeneration system, and the yield of 7β-OH-AD has also been improved (Fig. 4B).

Although PS can be utilized by *Mycolicibacterium* for growth and metabolism when there is no glucose [48], the growth of strains will be limited when the glucose concentration is insufficient in the fermentation medium. Moreover, glucose can be used as a cosubstrate for the regeneration of NADPH [49, 50]. Therefore, the concentration and feeding time of glucose were worth studying for the growth of *Mycolicibacterium* and the conversion of sterols (Fig. 5C and D). Finally, the highest yield of 7β-OH-AD was achieved under the cofactor recycling system and optimal fermentation conditions in M3-*mBM3*-*0*-*NADK2*-*G6PDH2*.

## Conclusions

This study aimed to develop an engineered *M. neoaurum* to realize the one-pot biotransformation from PS to 7β-OH-AD. The enzyme activity of mP450-BM3 was increased through protein engineering and the content of NADPH was well replenished by co-expressed NADK and G6PDH in the engineered *M. neoaurum*. Finally, the titer of 7β-OH-AD reached 164.52 mg L^− 1^ under optimal conditions. This study was the first to report the production of 7β-OH-AD from PS by microbial transformation. The efficient production of 7β-OH-AD with the coenzyme recycling and regeneration system was achieved for the first time. This work provided a design concept for engineered strains to produce high-value steroid compounds by microbial transformation in the pharmaceutical industry.

## Materials and methods

### Strains, plasmids, and culture conditions

All the strains and plasmids used in this study are listed in Table 1. *Escherichia coli* DH 5α (TransGen Biotech Co., Ltd. Beijing, China) was used for plasmid amplification.

The wild type *Mycolicibacterium* neoaurum ATCC 25,795 as a source of gene NADK and G6PDH was purchased from American Type Culture Collection (ATCC). The steroidal intermediate AD producer M3 was constructed by Xu [30]. The primers used for the construction of recombinant strains are described in Additional file 1: Table S1.

As a host for the molecular cloning of candidate genes, *E. coli* DH 5α was cultured at 37 °C and 220 rpm in liquid Luria-Bertani broth medium containing 50 mg L^− 1^ kanamycin for plasmid selection. The strains derived form *M. neoaurum* ATCC 25,795 were cultivated in a seed medium as described by Yao et al. [51]. After culturing in the seed medium for 36 h, the strains were then inoculated into the fermentation medium. The fermentation medium contains glucose 10 g L^− 1^, K_2_HPO_4_ 0.5 g L^− 1^, MgSO_4_ 0.5 g L^− 1^, (NH_4_)_2_HPO_4_ 3.5 g L^− 1^, Tween 80 7 g L^− 1^, citric acid 2 g L^− 1^, PS 3 g L^− 1^, and ammonium iron citrate 0.05 g L^− 1^ with a pH value of 7.2.

### Site-directed mutagenesis and construction of recombinant strains

The key amino acid residues of mP450-BM3 were analyzed by docking P450-BM3 with substrate AD in Autodock4.2 program suite. Site-directed mutagenesis of mP450-BM3 was constructed with a KOD-One site mutation kit from Toyobo Co. Ltd (Japan). The primers comprising required codons for mutations were designed (Additional file 1: Table S1). The P450-BM3 and mP450-BM3 with optimized codons for *Mycolicibacterium* were amplified with primers P1/P2 and then linked into the *E. coli*/*Mycolicibacterium* shuttle vector pMV261, which was successively digested by BamH I and EcoR I to form the recombinant plasmids pMV261-*BM3* and pMV261-*mBM3*. The plasmids pMV261-*mBM3*-*0* was constructed in the same way as before. The genes NADK and G6PDH from *M. neoaurum* were amplified with primers P3/P4 and P5/P6, and then linked to plasmid pMV261 to construct the recombinant plasmids pMV261-*NADK* and pMV261-*G6PDH*, respectively. The genes NADK and G6PDH containing ribosome binding site of pMV261 (designated as NADK2 and G6PDH2) were then amplified from recombinant plasmids pMV261-*NADK* and pMV261-*G6PDH* with primers P7/P8 and P9/P10, respectively. Finally, the genes NADK2 and G6PDH2 were respectively linked to the plasmid pMV261-*mBM3*-*0* to construct the recombinant plasmids pMV261-*mBM3*-*0*-*NADK2* and pMV261-*mBM3*-*0*-*G6PDH2*. The recombinant plasmid pMV261-*mBM3*-*0*-*NADK2-G6PDH2* was constructed on basis of plasmid pMV261-*mBM3*-*0*-*NADK2* with primers P11/P12.

The constructed recombinant plasmids were then electro-transformed into the strain M3. The empty vector pMV261 was also transformed into the M3 as a control. The obtained transformants were verified by PCR. The selected recombinants were then used for further characterization.

### Determination of enzyme activities of *7β*-hydroxylase, NADK and G6PDH

In this study, mP450-BM3 was mutated to form a sterol 7β-hydroxylase (mP450-BM3-0), and its enzyme activity was detected by measuring the formation of 7β-OH-AD with high-performance liquid chromatography (HPLC). The standard reaction mixture contained 50 mM Tris-HCL buffer (pH 7.0), cell-free extract, and 0.1 mM AD dissolved in 5% methanol. NADPH (0.5 mM) was added to the mixture to start the reaction and the mixture was incubated at 30 °C for 30 min. One unit (U) of 7β-hydroxylase activity is defined as the amount of enzyme required to convert 1 µM of AD into 7β-OH-AD per minute. The NAD kinase activity was assayed at 30 °C as described previously with a slight modification [52]. In brief, the formation of NADPH was continuously measured at A_340_ in a reaction mixture (1.0 mL) composed of 5.0 mM NAD^+^, 5.0 mM ATP, 5.0 mM MgCl_2_, 5.0 mM glucose-6-phosphate, 0.5 U glucose-6-phosphate dehydrogenase, 100 mM Tris/HCl (pH 8.0) and an appropriate amount of enzyme. One unit of NAD kinase activity is defined as the amount of NAD kinase required to produce 1.0 µmol NADPH within 1 min at 30 °C in an initial mixture (1.0 mL). For the G6PDH activity assay, the reaction mixture (2.56 mL) contained 70 mM Tris-HCL buffer (pH 7.2), 12 mM MgCL_2,_ 1 mM NADP^+^, 20 mM glucose-6-phosphate and 0.1 mL cell extract enzyme. One unit of G6PDH activity is defined as the amount of enzyme required to form 1 µM NADPH per minute [53].

### Optimization of biotransformation conditions

Firstly, the influence of the dosage of whole-cell biocatalyst (6%, 9%, 12% and 15%) on the conversion of PS into 7β-OH-AD was studied. Then, the effect of different substrate concentrations (1, 3, 5, and 7 g L^− 1^) on PS conversion was also investigated. Finally, the feeding time (3th, 4th, 5th, and 6th day) and concentration (10, 20, 30, and 40 g L^− 1^) of glucose were optimized to further improve 7β-OH-AD production.

### Extraction, separation, and analysis of products

The products converted from PS were extracted with ethyl acetate. The qualitative analysis and purification of the products were performed on TLC with HSGF254 plates (20 × 20 cm, Qingdao Marine Chemical Factory, China) with ethyl acetate/petroleum ether (3:2, v/v) as developing solvent. The purified products were used for subsequent structural identification by HRMS. HRMS was performed on a Micromass GCT instrument (Micromass UK, UK) via electron ionization (EI^+^) measurements under the condition: source temperature (250 °C) and electron energy (70 eV).

The gas chromatograph (GC) system (Agilent 7820 A, CA, USA) equipped with an HP-5 column (30 m × 0.25 mm, 0.25 μm film thickness) was used to determine the remaining content of phytosterol. The oven temperature was programmed as follows: 200 °C for 2 min, 200 to 280 °C within 4 min, 280 °C for 2 min, 280 to 305 °C within 1.5 min, and 305 °C for 10 min. Inlet temperature and flame-ionization detector temperature were maintained at 320 °C. The quantitative analysis of transformation products was carried out by HPLC (Agilent Technologies, Inc., Santa Clara, CA, USA) on a SDB C18 column (5 μm, 4.6 mm × 250 mm, Elite, China) at 30 °C with methanol/water (80:20, v/v) as the mobile phase with a flow rate of 1 mL min^− 1^. AD and 7β-OH-AD were detected by an ultraviolet spectrophotometry at 254 nm.

## Supplementary Information

**Additional file 1.** Additional figures and Table.Table S1. Primers used in this study. Figure S1. Gene cloning and identification of the recombinant plasmids. Figure S2. TLC chromatogram comparison of the products from the transformation of PS and AD by M3-261, M3-*BM3* and M3-*mBM3*, respectively. Figure S3. Qualitative and quantitative analysis of the products by engineered strains. Figure S4. 7β-OH-AD producing strains fermentation and detection. Figure S5. Relative activities of mutants S72W, V78L, A82M, A82L, T88S, A328G, A330W, A330P and mP450-BM3 (WT).

## Data Availability

All data generated and analyzed during this study are included in this published article and its supplementary information files.

## Abbreviations

PS: Phytosterols
AD: Androstene-3,17-dione
7β-OH-AD: *7β*-Hydroxyandrostene-3,17-dione
NADK: NAD kinase
G6PDH: Glucose-6-phosphate dehydrogenase
NADPH: Nicotinamide adenine dinucleotide phosphate
HP-β-CD: Hydroxypropyl-*β*-cyclodextrin

## References

[1] Fragkaki A, Angelis Y, Koupparis M, Tsantili-Kakoulidou A, Kokotos G, Georgakopoulos C (2009). Structural characteristics of anabolic androgenic steroids contributing to binding to the androgen receptor and to their anabolic and androgenic activities: applied modifications in the steroidal structure. Steroids.

[2] Donova MV, Egorova OV (2012). Microbial steroid transformations: current state and prospects. Appl Microbiol Biotechnol.

[3] Shoda J, Toll A, Axelson M, Pieper F, Wikvall K, Sjövall J (1993). Formation of *7α*-and *7β*‐hydroxylated bile acid precursors from 27‐hydroxycholesterol in human liver microsomes and mitochondria. Hepatology.

[4] Kimura A, Mahara R, Inoue T, Nomura Y, Murai T, Kurosawa T, Tohma M, Noguchi K, Hoshiyama A, Fujisawa T (1999). Profile of urinary bile acids in infants and children: developmental pattern of excretion of unsaturated ketonic bile acids and *7β*-hydroxylated bile acids. Pediatric Res.

[5] Le Mée S, Hennebert O, Ferrec C, Wülfert E, Morfin R (2008). *7β*-hydroxy-epiandrosterone-mediated regulation of the prostaglandin synthesis pathway in human peripheral blood monocytes. Steroids.

[6] Chau M, Jennewein S, Walker K, Croteau R (2004). Taxol biosynthesis: molecular cloning and characterization of a cytochrome P450 taxoid *7β*-hydroxylase. Chem Biol.

[7] Donova M (2022). Microbial steroid production technologies: current trends and prospects. Microorganisms.

[8] Tonin F, Arends IW (2018). Latest development in the synthesis of ursodeoxycholic acid (UDCA): a critical review. Beilstein J Org Chem.

[9] Eggert T, Bakonyi D, Hummel W (2014). Enzymatic routes for the synthesis of ursodeoxycholic acid. J Biotechnol.

[10] Loewenthal H (1959). Selective reactions and modification of functional groups in steroid chemistry. Tetrahedron..

[11] Pellissier H, Santelli M (2001). Chemical and biochemical hydroxylations of steroids: a review. Org Prep Proced Int.

[12] Manley JB, Anastas PT, Cue BW (2008). Frontiers in Green Chemistry: meeting the grand challenges for sustainability in R&D and manufacturing. J Cleaner Product.

[13] Rosłoniec KZ, Wilbrink MH, Capyk JK, Mohn WW, Ostendorf M, Van Der Geize R, Dijkhuizen L, Eltis LD (2009). Cytochrome P450 125 (CYP125) catalyses C26-hydroxylation to initiate sterol side‐chain degradation in *Rhodococcus jostii* RHA1. Mol Microbiol.

[14] Wang R, Sui P, Hou X, Cao T, Jia L, Lu F, Singh S, Wang Z, Liu X (2017). Cloning and identification of a novel steroid *11α*-hydroxylase gene from *Absidia coerulea*. J Steroid Biochem Molecular Biol.

[15] Chen J, Fan F, Qu G, Tang J, Xi Y, Bi C, Sun Z, Zhang X (2020). Identification of *Absidia orchidis* steroid *11β*-hydroxylation system and its application in engineering *Saccharomyces cerevisiae* for one-step biotransformation to produce hydrocortisone. Metab Eng.

[16] Holland HL, Thomas EM (1982). Microbial hydroxylation of steroids. 8. Incubation of Cn halo-and other substituted steroids with Cn hydroxylating fungi. Can J Chem.

[17] Pádua RM, Oliveira AB, Souza Filho JD, Takahashi JA, Braga FC (2007). Biotransformation of digitoxigenin by *Cochliobolus lunatus*. J Braz Chem Soc.

[18] Yildirim K, Kuru A, Küçükbaşol E (2020). Microbial transformation of androstenedione by *Cladosporium sphaerospermum* and *Ulocladium chartarum*. Biocatal Biotransform.

[19] Munro AW, Leys DG, McLean KJ, Marshall KR, Ost TW, Daff S, Miles CS, Chapman SK, Lysek DA, Moser CC (2002). P450 BM3: the very model of a modern flavocytochrome. Trends Biochem Sci.

[20] Cirino PC, Arnold FH (2003). A self-sufficient peroxide‐driven hydroxylation biocatalyst. Angew Chem Int Ed.

[21] Chen W, Fisher MJ, Leung A, Cao Y, Wong LL (2020). Oxidative diversification of steroids by nature-inspired scanning glycine mutagenesis of P450BM3 (CYP102A1). ACS Catal.

[22] Acevedo-Rocha CG, Gamble CG, Lonsdale R, Li A, Nett N, Hoebenreich S, Lingnau JB, Wirtz C, Fares C, Hinrichs H (2018). P450-catalyzed regio-and diastereoselective steroid hydroxylation: efficient directed evolution enabled by mutability landscaping. ACS Catal.

[23] Li A, Acevedo-Rocha CG, D’Amore L, Chen J, Peng Y, Garcia‐Borràs M, Gao C, Zhu J, Rickerby H, Osuna S (2020). Regio‐and stereoselective steroid hydroxylation at C7 by cytochrome P450 monooxygenase mutants. Angew Chem Int Ed.

[24] Donova MV, Dovbnya DV, Sukhodolskaya GV, Khomutov SM, Nikolayeva VM, Kwon I, Han K (2005). Microbial conversion of sterol-containing soybean oil production waste. J Chem Technol Biotechnol.

[25] Xiong L-B, Liu H-H, Zhao M, Liu Y-J, Song L, Xie Z-Y, Xu Y-X, Wang F-Q, Wei D-Z (2020). Enhancing the bioconversion of phytosterols to steroidal intermediates by the deficiency of kasB in the cell wall synthesis of *Mycobacterium neoaurum*. Microb Cell Fact.

[26] Whitehouse CJ, Bell SG, Wong L-L (2012). P450 BM3 (CYP102A1): connecting the dots. Chem Soc Rev.

[27] Richards L, Jarrold A, Bowser T, Stevens GW, Gras SL (2020). Cytochrome P450-mediated N-demethylation of noscapine by whole-cell biotransformation: process limitations and strategies for optimisation. J Ind Microbiol Biotechnol.

[28] Su L, Shen Y, Zhang W, Gao T, Shang Z, Wang M (2017). Cofactor engineering to regulate NAD^+^/NADH ratio with its application to phytosterols biotransformation. Microb Cell Fact.

[29] Zhao Y, Shen Y, Ma S, Luo J, Ouyang W, Zhou H, Tang R, Wang M (2019). Production of *5α-*androstene-3,17-dione from phytosterols by co-expression of *5α*-reductase and glucose-6-phosphate dehydrogenase in engineered *Mycobacterium neoaurum*. Green Chem.

[30] Wei W, Wang FQ, Fan SY, Wei DZ (2010). Inactivation and augmentation of the primary 3-ketosteroid-∆^1^-dehydrogenase in *Mycobacterium neoaurum* NwIB-01: biotransformation of soybean phytosterols to 4-androstene-3,17-dione or 1,4-androstadiene-3,17-dione. Appl Environ Microbiol.

[31] Shao M, Zhao Y, Liu Y, Yang T, Xu M, Zhang X, Rao Z (2019). Intracellular environment improvement of *Mycobacterium neoaurum* for enhancing androst-1,4-diene-3,17-dione production by manipulating NADH and reactive oxygen species levels. Molecules.

[32] Lee W-H, Park J-B, Park K, Kim M-D, Seo J-H (2007). Enhanced production of ɛ-caprolactone by overexpression of NADPH-regenerating glucose 6-phosphate dehydrogenase in recombinant *Escherichia coli* harboring cyclohexanone monooxygenase gene. Appl Microbiol Biotechnol.

[33] Xiong LB, Liu HH, Xu LQ, Wei DZ, Wang FQ (2017). Role identification and application of SigD in the transformation of soybean phytosterol to *9α*-hydroxy-4-androstene-3, 17-dione in *Mycobacterium neoaurum*. J Agric Food Chem.

[34] Wang ZF, Huang YL, Rathman JF, Yang ST (2002). Lecithin-enhanced biotransformation of cholesterol to androsta‐1,4‐diene‐3,17‐dione and androsta‐4‐ene‐3,17‐dione. J Chem Technol Biotechnol.

[35] Grobe S, Badenhorst CP, Bayer T, Hamnevik E, Wu S, Grathwol CW, Link A, Koban S, Brundiek H, Großjohann B (2021). Engineering regioselectivity of a P450 monooxygenase enables the synthesis of ursodeoxycholic acid via *7β*-hydroxylation of lithocholic acid. Angew Chem Int Ed.

[36] Sallam L, Osman M, Hamdy A, Zaghlol GM (2008). Microbial transformation of phytosterols mixture from rice bran oil unsaponifiable matter by selected bacteria. World J Microbiol Biotechnol.

[37] Tang R, Ren X, Xia M, Shen Y, Tu L, Luo J, Zhang Q, Wang Y, Ji P, Wang M (2021). Efficient one-step biocatalytic multienzyme cascade strategy for direct conversion of phytosterol to C-17-hydroxylated steroids. Appl Environ Microbiol.

[38] Xie R, Shen Y, Qin N, Wang Y, Su L, Wang M (2015). Genetic differences in ksdD influence on the ADD/AD ratio of *Mycobacterium neoaurum*. J Ind Microbiol Biotechnol.

[39] Vail RB, Homann MJ, Hanna I, Zaks A (2005). Preparative synthesis of drug metabolites using human cytochrome P450s 3A4, 2C9 and 1A2 with NADPH-P450 reductase expressed in *Escherichia coli*. J Ind Microbiol Biotechnol.

[40] Bernhardt R, Urlacher VB (2014). Cytochromes P450 as promising catalysts for biotechnological application: chances and limitations. Appl Microbiol Biotechnol.

[41] Xu LH, Du YL (2018). Rational and semi-rational engineering of cytochrome P450s for biotechnological applications. Synthetic and systems biotechnology.

[42] Lutz S (2010). Beyond directed evolution-semi-rational protein engineering and design. Curr Opin Biotechnol.

[43] Wang M, Chen B, Fang Y, Tan T (2017). Cofactor engineering for more efficient production of chemicals and biofuels. Biotechnol Adv.

[44] Heux S, Cachon R, Dequin S (2006). Cofactor engineering in *Saccharomyces cerevisiae*: expression of a H_2_O-forming NADH oxidase and impact on redox metabolism. Metab Eng.

[45] Papapetridis I, van Dijk M, Dobbe A, Metz B, Pronk JT, van Maris AJ (2016). Improving ethanol yield in acetate-reducing *Saccharomyces cerevisiae* by cofactor engineering of 6-phosphogluconate dehydrogenase and deletion of ALD6. Microb Cell Fact.

[46] Su L, Shen Y, Gao T, Luo J, Wang M (2017). Improvement of AD biosynthesis response to enhanced oxygen transfer by oxygen vectors in *Mycobacterium neoaurum* TCCC 11979. Appl Biochem Biotechnol.

[47] Szentirmai A (1990). Microbial physiology of side chain degradation of sterols. J Ind Microbiol.

[48] Zhao A, Zhang X, Li Y, Wang Z, Lv Y, Liu J, Alam MA, Xiong W, Xu J (2021). *Mycolicibacterium* cell factory for the production of steroid-based drug intermediates. Biotechnol Adv.

[49] Wu Y, Li H, Zhang XM, Gong JS, Li H, Rao ZM, Shi JS, Xu ZH (2015). Improvement of NADPH-dependent P450-mediated biotransformation of *7α*,*15α*-diOH-DHEA from DHEA by a dual cosubstrate-coupled system. Steroids.

[50] Siedler S, Bringer S, Bott M (2011). Increased NADPH availability in *Escherichia coli*: improvement of the product per glucose ratio in reductive whole-cell biotransformation. Appl Microbiol Biotechnol.

[51] Yao K, Xu LQ, Wang FQ, Wei DZ (2014). Characterization and engineering of 3-ketosteroid-∆^1^-dehydrogenase and 3-ketosteroid-*9α*-hydroxylase in *Mycobacterium neoaurum* ATCC 25795 to produce *9α*-hydroxy-4-androstene-3,17-dione through the catabolism of sterols. Metab Eng..

[52] Kawai S, Mori S, Mukai T, Suzuki S, Yamada T, Hashimoto W, Murata K (2000). Inorganic polyphosphate/ATP-NAD kinase of *Micrococcus flavus* and *Mycobacterium tuberculosis* H37Rv. Biochem Biophys Res Commun.

[53] Shao M, Zhang X, Rao Z, Xu M, Yang T, Li H, Xu Z, Yang S (2016). Efficient testosterone production by engineered *Pichia pastoris* co-expressing human *17β*-hydroxysteroid dehydrogenase type 3 and *Saccharomyces cerevisiae* glucose 6-phosphate dehydrogenase with NADPH regeneration. Green Chem..

