# Supplementary material for: One-pot biosynthesis of 7β-hydroxyandrost-4-ene-3,17-dione from phytosterols by cofactor regeneration system in engineered mycolicibacterium neoaurum
Source: Microb Cell Fact. 2022 Apr 9;21:59. doi: 10.1186/s12934-022-01786-5 (PMC8994266; doi:10.1186/s12934-022-01786-5)
Supplement: Supplementary file 1 — Additional file 1. Additional figures and Table.Table S1. Primers used in this study. Figure S1. Gene cloning and identification of the recombinant plasmids. Figure S2. TLC chromatogram comparison of the products from the transformation of PS and AD by M3-261, M3-BM3 and M3-mBM3, respectively. Figure S3. Qualitative and quantitative analysis of the products by engineered strains. Figure S4. 7β-OH-AD producing strains fermentation and detection. Figure S5. Relative activities of mutants S72W, V78L, A82M, A82L, T88S, A328G, A330W, A330P and mP450-BM3 (WT). [file 12934_2022_1786_MOESM1_ESM.docx]

**Supporting Information**

**One-pot biosynthesis of *7β*-hydroxyandrost-4-ene-3,17-dione from phytosterols by cofactor regeneration system** **in engineered *Mycolicibacterium neoaurum***

Yun-Qiu Zhao†, Yong-Jun Liu†, Wei-Ting Ji, Kun Liu, Bei Gao, Xin-Yi Tao, Ming Zhao*, Feng-Qing Wang* and Dong-Zhi Wei

State Key Laboratory of Bioreactor Engineering, Newworld Institute of Biotechnology, East China University of Science and Technology, Shanghai, China, 200237

*Address correspondence to

Ming Zhao: zhaom@ecust.edu.cn

Feng-Qing Wang: fqwang@ecust.edu.cn

**Table S1.** Primers used in this study

| **Name** | **Description** | **Application** |
| --- | --- | --- |
| **Primers 5’-3’** |  |  |
| P1 | CGCGGATCCATGACCATCAAGGAGATGCC (*Bam*H I) | Cloning of gene (m)P450-BM3 |
| P2 | CCGGAATTCTTAGCCCGCCCAGACGTCCT (*Eco*R I) |  |
| P3 | CCGGAATTCATGACATTGCCACCGCGGGC (*Eco*R I) | Cloning of gene NADK |
| P4 | CCCAAGCTTTCAGCGCAGCAGCCGCAACC (*Hin*d III) |  |
| P5 | CCGGAATTCATGAGCACAGCCGAGGCA (*Eco*R I) | Cloning of gene G6PDH |
| P6 | CCCAAGCTTTCACGGCCGCCGCCACTC (*Hin*d III) |  |
| P7 | CCGGAATTCTAAGTAGCGGGGTTGCCGTCA (*Eco*R I) | Cloning of gene NADK2 |
| P8 | CCCAAGCTTTCAGCGCAGCAGCCGCAACC (*Hin*d III) |  |
| P9 | CCGGAATTCTAAGTAGCGGGGTTGCCGTCA (*Eco*R I) | Cloning of gene G6PDH2 |
| P10 | CCCAAGCTTTCACGGCCGCCGCCACTC (*Hin*d III) |  |
| P11 | CCCAAGCTTGGTGACCACAACGACGCG (*Hin*d III) | Cloning of gene G6PDH2 with |
| P12 | CCGGTTAACTCACGGCCGCCGCCACTCG (*Hp*a I) | promoter hsp60 |
| S72W-F | CTC**TGG**CAGGCGCTCAAGTACCTGCGGGAC |  |
| S72W-R | GTTCTTGTCGAACCGCGACTCGTC |  |
| V78L-F | TAC**CTG**CGGGACATCCTCGGCGACGGCCTC |  |
| V78L-R | CTTGAGCGCCTGCCAGAGGTTCTT |  |
| A82L-F | ATC**CTC**GGCGACGGCCTCGCGTCGTCGTGG |  |
| A82L-R | GTCCCGCAGGTACTTGAGCGCCTG | mP450-BM3 key mutation sites |
| T88S-F | GGC**TCG**TCGTGGACCCACGAGAAGAACTGG |  |
| T88S-R | GAGGCCGTCGCCGAGGATGTCCCG |  |
| A328G-F | ACC**GGC**CCGTGGTTCTCGCTGTACGCCAAG |  |
| A328G-R | GGGCCACAGCCGGAGCGCCTCGTT |  |
| A330W-F | CCG**TGG**TTCTCGCTGTACGCCAAGGAGGAC |  |
| A330W-R | GCCGGTGGGCCACAGCCGGAGCGC |  |

**Notes**: The restriction enzyme sites were underlined; the mutation sites were bold.

**
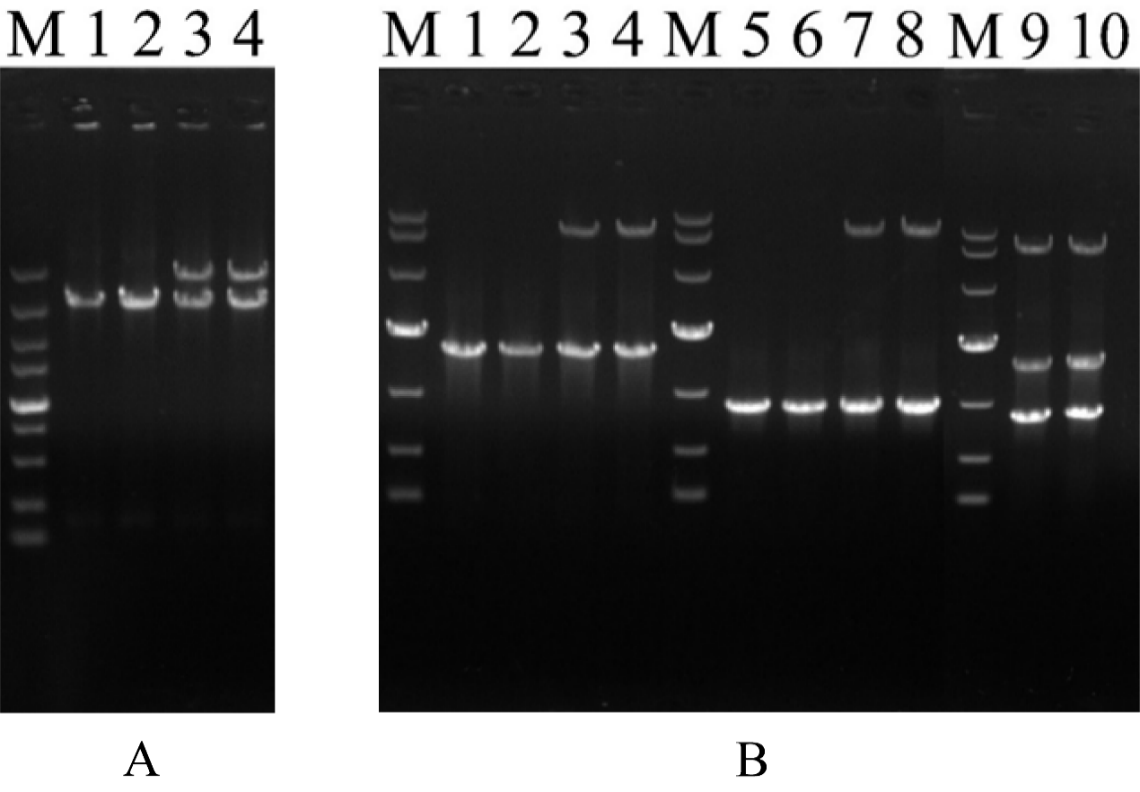
**

**Fig. S1** Gene cloning and identification of the recombinant plasmid. **A** Construction of the recombinant plasmid pMV261-*mBM3* for 7β-hydroxylase production in *E. coli* DH5α. The length of mP450-BM3 gene from *Bacillus megaterium* is 3150 bp. The length of pMV261 is 4488 bp. M, DL5000 marker; lane 1-2, the amplified mP450-BM3 gene by PCR; lane 3-4, the recombinant plasmid pMV261-*mBM3* digested by *Bam*H I/*Eco*R I. **B** Construction of the recombinant plasmids pMV261-*mBM3*-*0*-*G6PDH2*, pMV261-*mBM3*-*0*-*NADK2* and pMV261-*mBM3-0*-*NADK2*-*G6PDH2* for G6PDH2 and NADK2 production respectively in *E. coli* DH 5α. The length of G6PDH2 and NADK2 genes are 1647 bp and 996 bp respectively. The length of pMV261-*mBM3*-*0* is 7630 bp. M, DL10000 marker; lane 1-2, the amplified G6PDH2 gene by PCR; lane 3-4, the recombinant plasmid pMV261-*mBM3*-*0*-*G6PDH2* digested by *Eco*R I/*Hin*d III; lane 5-6, the amplified NADK2 gene by PCR; lane 7-8, the recombinant plasmid pMV261-*mBM3*-*0*-*NADK2* digested by EcoR I/Hind III; lane 9-10, the recombinant plasmid pMV261-*mBM3-0*-*NADK2*-*G6PDH2* digested by *Hin*d III/*Hp*a I.


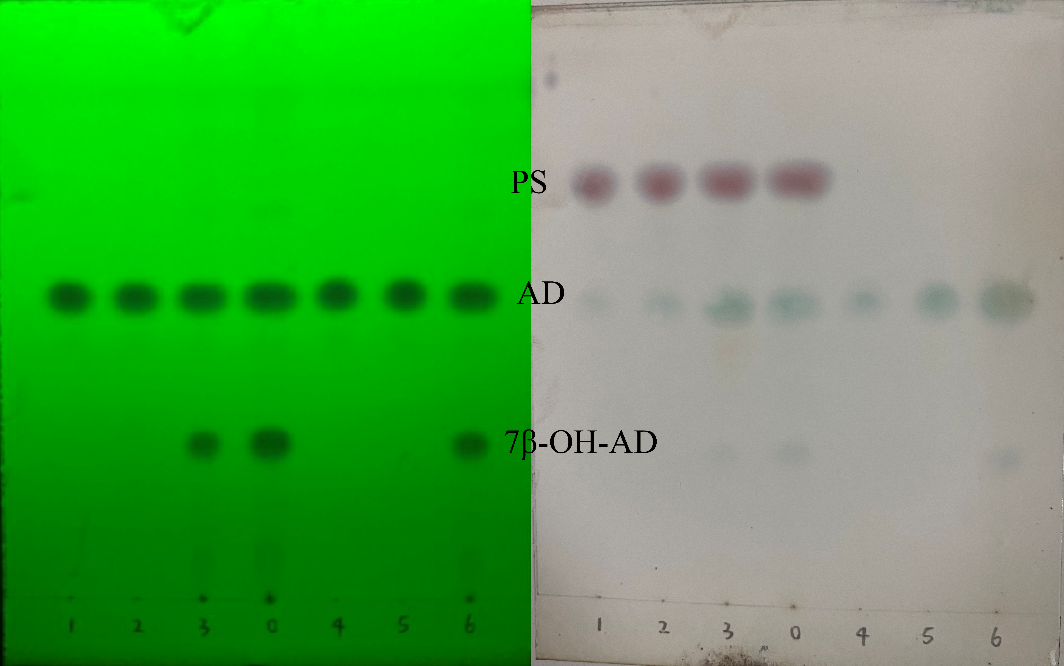


**Fig. S2** TLC chromatogram comparison of the products from the transformation of PS and AD by M3-261, M3*-BM3* and M3*-mBM3*, respectively. Lane 0, standards of PS, AD and 7β-OH-AD. Lane 1-3, biotransformation of PS by M3-261, M3*-BM3* and M3*-mBM3*, respectively. Lane 4-6, biotransformation of AD by M3*-*261, M3*-BM3* and M3*-mBM3*, respectively.


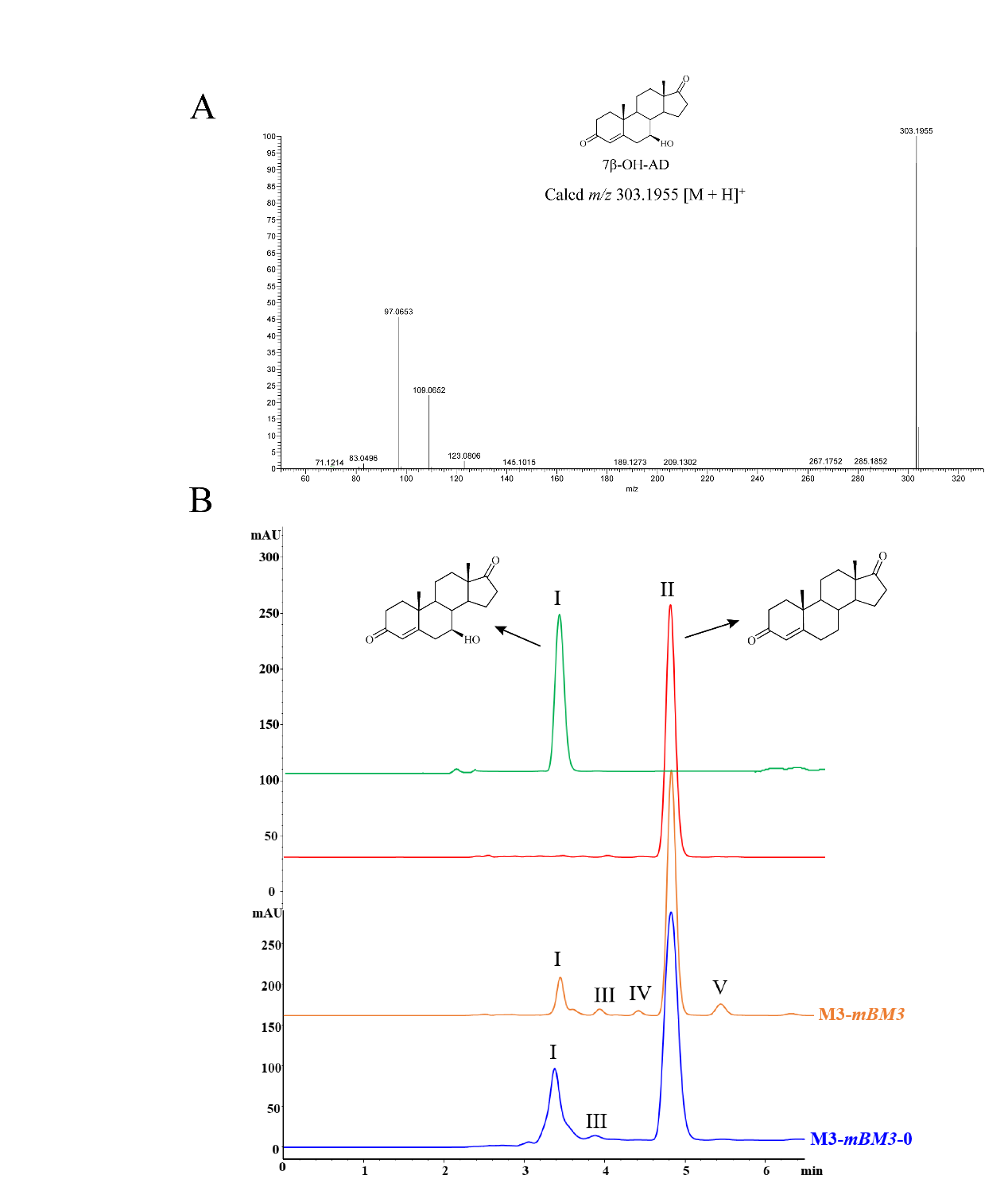


**Fig. S3** Qualitative and quantitative analysis of the products by engineered strains. **A** HRMS analysis of the purified product. Mass spectra showed that the purified product with *m/z* was 303.19 at [M+H] ^+^, which indicated the molecular weight of purified product was 302, consistent with molecular weight of compound C_19_H_26_O_3_. **B** HPLC chromatogram comparison of the products from the transformation of PS by M3-261 (purple), M3*-mBM3* (orange) and M3-*mBM3*-*0* (blue). Peak I, standard of 7β-OH-AD. Peak II, standard of AD. Peak III, IV and V were by-products.


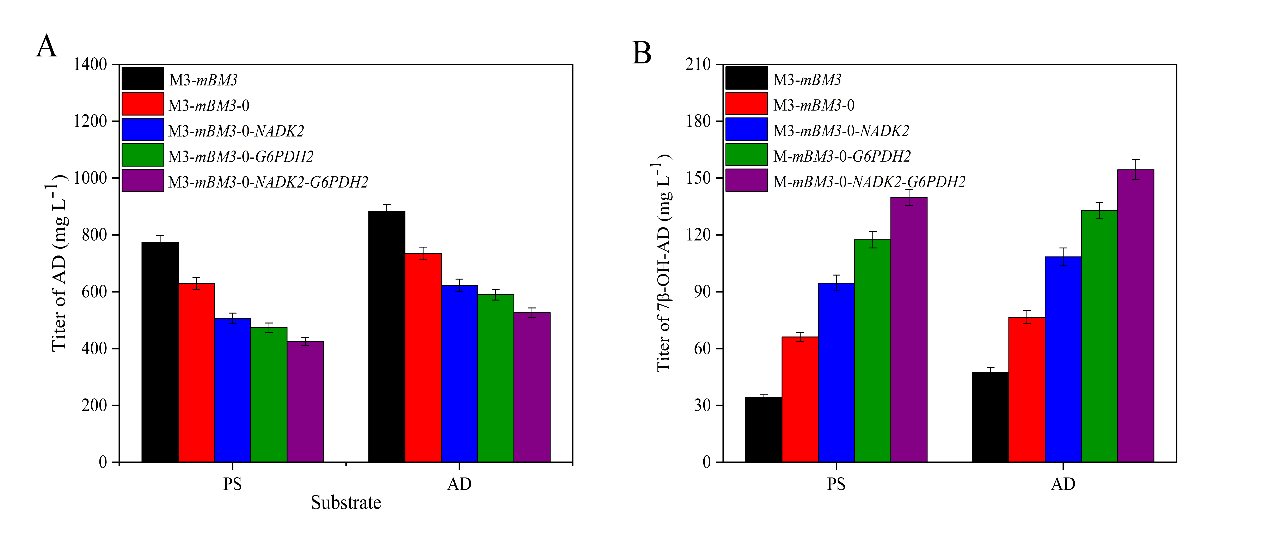


**Fig. S4** 7β-OH-AD producing strains fermentation and detection**. A** The remaining titer of AD from the transformation of PS and AD by different recombinant *M.* *neoaurum* strains. **B** The titer of 7β-OH-AD from the transformation of PS and AD by different recombinant *M.* *neoaurum* strains. All assays were performed in triplicate with three independent measurements. Standard deviations of the biological replicates are represented by error bars.





**Fig. S5** Relative activity of mutants S72W, V78L, A82M, A82L, T88S, A328G, A330W, A330P and mP450-BM3 (WT). All assays were performed in triplicate with three independent measurements. Standard deviations of the biological replicates are represented by error bars.
